# Five Fatty Acyl-Coenzyme A Reductases Are Involved in the Biosynthesis of Primary Alcohols in *Aegilops tauschii* Leaves

**DOI:** 10.3389/fpls.2017.01012

**Published:** 2017-06-12

**Authors:** Meiling Wang, Hongqi Wu, Jing Xu, Chunlian Li, Yong Wang, Zhonghua Wang

**Affiliations:** ^1^State Key Laboratory of Crop Stress Biology for Arid Areas, College of Agronomy, Northwest A&F UniversityYangling, China; ^2^Guizhou Rapeseed Institute, Guizhou Academy of Agricultural SciencesGuiyang, China

**Keywords:** fatty acyl-coenzyme A reductase, primary alcohol, cuticular wax, *Saccharomyces cerevisiae*, *Aegilops tauschii*, *Triticum aestivum*

## Abstract

The diploid *Aegilops tauschii* is the D-genome donor to hexaploid wheat (*Triticum aestivum*) and represents a potential source for genetic study in common wheat. The ubiquitous wax covering the aerial parts of plants plays an important role in protecting plants against non-stomatal water loss. Cuticular waxes are complex mixtures of very-long-chain fatty acids, alkanes, primary and/or secondary alcohols, aldehydes, ketones, esters, triterpenes, sterols, and flavonoids. In the present work, primary alcohols were identified as the major components of leaf cuticular wax in *Ae. tauschii*, with C26:0-OH being the dominant primary alcohol. Analysis by scanning electron microscope revealed that dense platelet-shaped wax crystals were deposited on leaf surfaces of *Ae. tauschii*. Ten putative wax biosynthetic genes encoding fatty acyl-coenzyme A reductase (FAR) were identified in the genome of *Ae. tauschii*. Five of these genes, *Ae.tFAR1*, *Ae.tFAR2*, *Ae.tFAR3*, *Ae.tFAR4*, and *Ae.tFAR6*, were found expressed in the leaf blades. Heterologous expression of the five Ae.tFARs in yeast (*Saccharomyces cerevisiae*) showed that Ae.tFAR1, Ae.tFAR2, Ae.tFAR3, Ae.tFAR4, and Ae.tFAR6 were predominantly responsible for the accumulation of C16:0, C18:0, C26:0, C24:0, and C28:0 primary alcohols, respectively. In addition, nine *Ae.tFAR* paralogous genes were located on D chromosome of wheat and the wheat nullisomic–tetrasomic lines with the loss of *Ae.tFAR3* and *Ae.tFAR4* paralogous genes had significantly reduced levels of primary alcohols in the leaf blades. Collectively, these data suggest that *Ae.tFAR1*, *Ae.tFAR2*, *Ae.tFAR3*, *Ae.tFAR4*, and *Ae.tFAR*6 encode alcohol-forming FARs involved in the biosynthesis of primary alcohols in the leaf blades of *Ae. tauschii*. The information obtained in *Ae. tauschii* enables us to better understand wax biosynthesis in common wheat.

## Introduction

The outermost surfaces of land plants are covered by the cuticle, which is composed of a cutin polymer matrix and cuticular waxes. Cutin is a three dimensional polymer of mainly C16 and C18 hydroxy fatty acids, which are cross-linked in ester bonds directly or through a glycerol backbone (Pollard et al., 2008; Li and Beisson, 2009). Cuticular wax is a mixture of lipids, consisting of, for example, very-long-chain fatty acids (VLCFAs, C20 to C34) and their derivatives, including alcohols, aldehydes, alkanes, ketones, and wax esters (Kunst and Samuels, 2003; Samuels et al., 2008; Lee and Suh, 2013). Cutin and waxes are integrated into the outer portion of the polysaccharide cell wall, forming a lipid-impregnated zone known as the cuticular layer, and are also layered on top of the cuticular layer to create a hydrophobic surface devoid of polysaccharides called the cuticle proper (Jeffree, 2006). Cuticular wax has multiple functions in plants, with the primary one being as a water permeability barrier that restricts non-stomatal water loss from epidermal surfaces (Kerstiens, 2006; Domínguez et al., 2011). Cuticular wax also protects plants against ultraviolet radiation, pathogen attack, and insect destruction (Eigenbrode and Espelie, 1995; Jenks et al., 2002; Solovchenko and Merzlyak, 2003; Pfündel et al., 2006).

Wax composition can be quite distinct depending on species, organs, and developmental stage. The composition also tends to be influenced by a variety of environmental factors, such as light, temperature and humidity (Kolattukudy, 1996; Samuels et al., 2008). The biosynthesis of wax has been thoroughly characterized in Arabidopsis. It starts with the *de novo* synthesis of C16 or C18 fatty acid in the plastids of epidermal cells. Then, elongation of C16 and C18 fatty acyl-CoAs via fatty acid elongase (FAE) complex in the endoplasmic reticulum (ER) leads to the production of VLCFA wax precursors (Samuels et al., 2008; Kunst and Samuels, 2009). Following elongation is the production of wax components by converting long-chain fatty acyl-CoAs via two different pathways: the acyl-reduction pathway, producing primary alcohols and wax esters (Samuels et al., 2008), and the decarbonylation pathway, generating aldehydes, alkanes, secondary alcohols, and ketones (Schneider-Belhaddad and Kolattukudy, 2000). Fatty acyl reductase catalyzes the conversion of fatty acyl to primary alcohols and different fatty acyl-CoA reductases (FARs) generally show distinct specificities for acyl chain lengths, leading to the production of fatty alcohols with various chain lengths. In Arabidopsis, FAR2/MS2 and FAR6 preferentially produce sporopollenin-related long-chain fatty alcohols (Doan et al., 2009; Chen et al., 2011). FAR1, FAR4, and FAR5 are primarily responsible for generating suberin-associated C22:0-OH, C20:0-OH, and C18:0-OH, respectively (Domergue et al., 2010), while FAR3/CER4 in Arabidopsis is associated with the production of C24:0, C26:0, C28:0, and C30:0 primary fatty alcohols (Rowland et al., 2006). Some FAR genes related to wax biosynthesis have subsequently been identified and characterized in bread wheat. Expression of these *TaFARs* in various heterologous systems leads to the accumulation of C18:0-OH to C30:0-OH. TaFAR3 catalyzes the biosynthesis of C28 primary alcohol, which is the major wax component in wheat leaf, whereas TaFAR1 and TaFAR4 generate C22 and C24 primary alcohols, respectively (Wang et al., 2015b, 2016).

In wheat, one of the key staple crops worldwide, primary alcohols of C20 to C32 are important cuticular wax components (Wang et al., 2015a). In a previous work, 32 genes were identified encoding FARs associated with wax biosynthesis in the leaf blade of wheat and 12 of them are located on D genome (Wang et al., 2016). Of 12 genes, *TaFAR2* and *TaFAR5* are responsible for the biosynthesis of C18 and C22 primary alcohols, respectively (Wang et al., 2015c, 2016). However, information regarding the rest of genes encoding alcohol-forming enzymes on D genome is still lacking. Common wheat is an allohexaploid species (*Triticum aestivum*, 2*n* = 42, AABBDD), deriving from the hybridization between tetraploid wheat *Triticum turgidum* (2*n* = 28, AABB) and diploid grass *Aegilops tauschii* (2*n* = 14, DD) (Pestsova et al., 2000; Jia et al., 2013). Being the D-genome donor to hexaploid wheat, *Ae. tauschii* serves as an important resource for wheat breeding and provides ample information for genomic study in wheat. The availability of complete genome data makes the utility of this species even further (Jia et al., 2013). Thus, taking advantage of *Ae. tauschii*, we may have a better understanding of mechanisms of primary alcohol biosynthesis in wheat. The objective of our present work was to identify FARs involved in the biosynthesis of wax primary alcohols in the leaf blades of *Ae. tauschii* and through analysis of these FARs to acquire more information on alcohol-forming FARs in hexaploid wheat.

## Materials and Methods

### Plant Materials and Growth Conditions

The wild diploid grass *Ae. tauschii*, hexaploid wheat (*T. aestivum* L.) cv. Chinese Spring (CS) and wheat nullisomic–tetrasomic lines were grown at the experimental farm of Northwest A&F University, Yangling, China. Eight-week old leaves at the seedling stage and flag leaves, leaf sheaths, peduncles, glumes, and anthers (160 days old) at the flowering stage were used for analysis of wax composition and micromorphology. Simultaneously, materials were collected for analysis of tissue-specific expression patterns of *Ae.tFARs*. The seedling leaves and flag leaves were used for quantitative real-time PCR (qRT-PCR) and cuticular wax analysis in wild-type wheat and nullisomic–tetrasomic lines.

### Wax Extraction and Chemical Characterization

Wax load and chemical composition were determined in *Ae. tauschii* and hexaploid wheat. Various organs including seedling leaves, flag leaves, leaf sheaths, peduncles, glumes, and anthers from *Ae. tauschii* and leaves from wheat were harvested and immersed in chloroform for 60 s to extract waxes. For analysis by GC-MS and GC-FID, dried wax samples were derivatized with equal amounts of pyridine (Alfa Aesar) and BSTFA [bis-N,O-(trimethylsilyl)trifluoroacetamide] (Fluka) for 40 min at 70°C in a small GC vials. Then, samples were dried under nitrogen gas before being re-dissolved in chloroform (Wang et al., 2015c). *n*-tetracosane (C24) was added as an internal standard. Wax compounds were quantified by gas chromatography equipped with mass spectrometric detector (GC-MS) (GCMS-QP2010, SHIMADZU, Japan). Each wax component load was calculated based on peak area of each compound and that of the C24 internal standard. The sample surface areas were determined using ImageJ software.

### Scanning Electron Microscopy (SEM) Analysis

To observe the micromorphology of wax crystals, various organs including seedling leaves, flag leaves, leaf sheaths, peduncles, glumes, and anthers were gently flattened and dried in oven. Sample segments were carefully mounted onto scanning electron microscopy (SEM) stubs, and then coated with superfine platinum powder in an ion sputter coater (Hitachi E-1045, Japan). A Hitachi S-4800 field emission SEM was used to view the surfaces of coated samples at an accelerating voltage of 5 kV (Wang et al., 2016).

### Isolation of *Ae.tFAR* Genes from *Ae. tauschii* Leaf

Total RNA was extracted from seedling leaves of wild *Ae. tauschii* using Trizol extraction reagent (TaKaRa, Japan). Then, 1 μg RNA was used for first-strand cDNA synthesis by PrimeScript^®^ reverse transcriptase (TaKaRa) with primer oligo (dT)18. To amplify the putative *Ae.tFAR*s, PCR was performed with HS PrimeSTAR^®^ DNA polymerase (TaKaRa) and the following cycling conditions: initial denaturation at 94°C for 1 min, followed by 35 cycles, 98°C for 10 s, 58°C for 30 s, 72°C for 2 min. Gene-specific primers Ae.tFARx were used to amplify the full-length coding sequences (Supplementary Table 1). The amplified DNA fragments were separated and purified using the DNA purification kit (TIANGEN, China). These corresponding fragments were cloned into pMD-18T vector (TaKaRa) and sequenced. Subsequent construction of expression vectors was made using this clone as template.

### Functional Expression of Ae.tFARs in Yeast

The entire coding sequences of *Ae.tFAR1*, *Ae.tFAR2*, *Ae.tFAR3*, *Ae.tFAR4*, and *Ae.tFAR6* were amplified using primers shown in Supplementary Table 1. The amplified DNA fragments were double digested and cloned into the corresponding sites of the yeast (*Saccharomyces cerevisiae*) expression vector pYES3 (Invitrogen) under the control of GAL1 promoter to generate pYES3-Ae.tFARx. A vector of p416 MET25-FLAG3:Sur4-F262A/K266L was used to obtain the yeast mutant INVSur4# (a mutation in ketoacyl-CoA synthase, KCS), which allows for the production of fatty acids with longer chain lengths in yeast (Denic and Weissman, 2007). The p416 MET25-FLAG3:Sur4-F262A/K266L was co-transformed with pYES3-Ae.tFARx into wild-type INVSc1 strain (*MATa his3-D1 leu2 trp1-289 ura3-52*). The corresponding empty vectors were used as positive control as detailed in Supplementary Table 2.

The yeast transformation was carried out as described by Gietz and Woods (2002). Yeast cells transformed with different vector combinations were cultured on synthetic complete (SC) selection medium lacking tryptophan and uracil with 2% (w/v) glucose for 48 h, induced by 2% (w/v) galactose for 12 h and followed by incubation in 0.1 M potassium phosphate and 2% (w/v) glucose for 24 h at 28°C. For extraction of lipids from yeast cells, 40 mL of liquid cultures were collected, re-suspended in 20% KOH/50% ethanol, and extracted three times with hexane at 70°C for 5 min as described by Wang et al. (2011).

For analysis of fatty alcohols, lipid mixtures from yeast were separated and purified by thin layer chromatography (TLC) plate (5 cm × 10 cm, silica gel, HSGF254, 0.15–0.20 mm) using chloroform as the mobile phase. Different lipids on the plates were then stained with primuline and observed by UV light. The fatty alcohol bands were scratched off and were further analyzed by GC-MS and GC-FID.

### Quantitative Real-Time PCR Analysis

Transcript levels were determined by qRT-PCR. Various tissues including seedling leaves, flag leaves, leaf sheaths, peduncles, glumes, and anthers from *Ae. tauschii* and seedling leaves and flag leaves from wheat were used for total RNA extractions. The RNA samples were then used for cDNA synthesis by PrimeScript^®^ reverse transcriptase following standard protocols. The qRT-PCR was performed using SYBR^®^ Premix Ex Taq^TM^ (TaKaRa) with gene-specific primers (Supplementary Table 1). *Ae. tauschii GAPDH* gene (GenBank accession no. KD518701) and wheat *Actin* gene (GenBank accession no. DQ115883) were used as internal expressed controls. Primers of *GAPDH* and *Actin* were designed to amplify a 177- and 118-bp fragment, respectively. Real-time PCR was carried out in 25 μl reaction volume using a CFX96 real-time PCR detection system (BIO-RAD, United States) with the following program: 1 cycle of 30 s at 95°C, followed by 40 cycles of 5 s at 95°C and 30 s at 60°C.

### Phylogenetic Analysis

Multiple sequence alignments were performed with DNAMAN program using amino acid sequences of FARs from different species. A phylogenetic tree was created with the MEGA software (version 5.1) based on a Maximum Likelihood method (1,000 replicates). TMHMM Server (version 2.0) was used to predict potential transmembrane domains.

## Results

### Chemical Composition and Micromorphology of Wax in the Leaf Blades of *Ae. tauschii*

**FIGURE 1:**
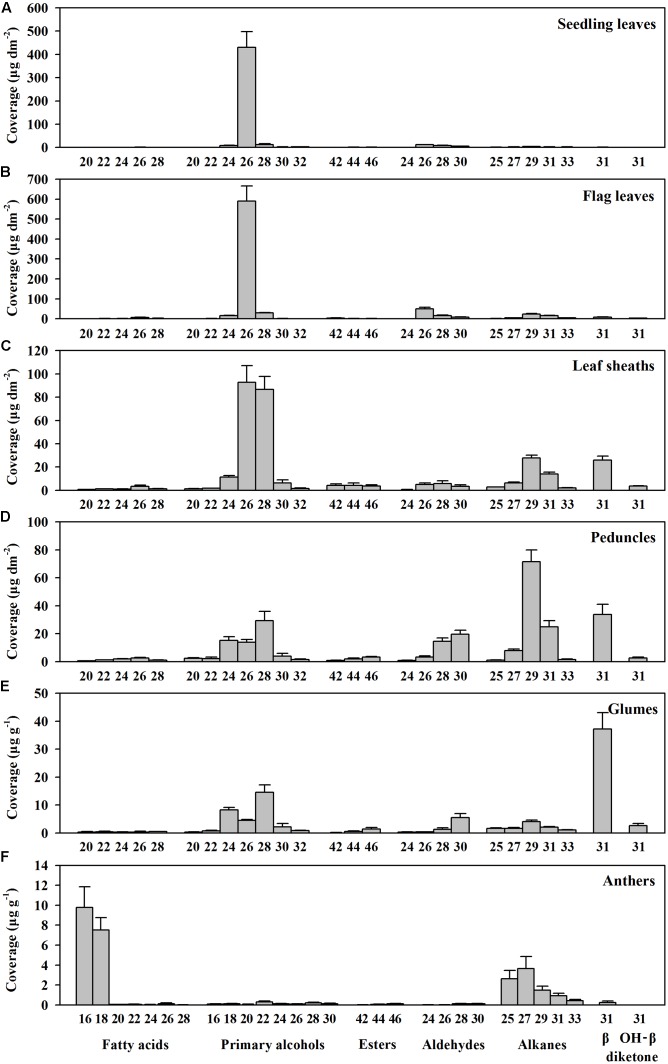
Chain length distributions within compound classes of cuticular wax from various organs of *Aegilops tauschii*. Cuticular wax mixtures were extracted from **(A)** seedling leaves, **(B)** flag leaves, **(C)** leaf sheaths, **(D)** peduncles, **(E)** glumes, and **(F)** anthers of *Ae. tauschii*. The coverage of individual compound is expressed as μg per unit dm^2^ (seedling leaves, flag leaves, leaf sheaths, and peduncles) and μg per unit g fresh weight (glumes and anthers). Values are means of three biological replicates. Error bars = SD.

To investigate genes involved in wax biosynthesis in *Ae. tauschii*, we first analyzed the wax load and composition from various organs, including seedling leaves, flag leaves, leaf sheaths, peduncles, glumes, and anthers by GC-MS and GC-FID. The results showed that the waxes from different organs consisted of the same chemical classes, including fatty acids, primary alcohols, esters, aldehydes, alkanes, hentriacontane-14, 16-dione (β-diketone), and OH-β-diketone (**Table 1**), however, the relative amount of each chemical class was quite different depending on organs. Alkanes and β-diketones were the dominant chemical species in panicle and glume, respectively, while fatty acids and aldehydes were major chemical classes in anthers. Primary alcohol was found to be the richest chemical class in leaves, accounting for 84% of total wax load in seedling leaves and 76% in flag leaves, whereas only small amounts of alkanes and aldehydes were detected in leaves (**Figure 1** and **Table 1**). The high level of primary alcohol was also observed in leaf sheaths, being 57% of total wax load (**Table 1**). The chain lengths of primary alcohols ranged from C20 to C32, among which C26 was the most prevalent one in seedling leaf, flag leaf, and leaf sheath (**Figures 1A–C**). Intermediate levels of primary alcohols, β-diketone and alkanes were observed. Chain length distributions of primary alcohols in peduncles and glumes varied from C20 to C32 and the dominant chain length was C28, which is different from that in leaves (**Figures 1D,E**). These results suggest that primary alcohols are the key wax components and vary considerably depending on organs in leaves of *Ae. tauschii*.

**Table 1:** Wax composition of seedling leaves, flag leaves, leaf sheaths, peduncles, glumes and anthers of in *Aegilops tauschii*.

Chemical class	Measurement	Seedling leaves	Flag leaves	Leaf sheaths	Peduncles	Glumes	Anthers
Total		544.4 ± 68.9	841.7 ± 80.8	356.2 ± 11.9	291.6 ± 37.5	104.5 ± 16.5	32.7 ± 4.4
Fatty acids	Amount	4.4 ± 0.3	12.8 ± 3.1	7.6 ± 1.0	7.3 ± 1.0	1.8 ± 0.6	17.6 ± 2.6
	Percentage	0.8 ± 0.1	1.5 ± 0.3	2.1 ± 0.3	2.5 ± 0.1	1.8 ± 0.8	53.9 ± 6.1
Prim. alcohols	Amount	457.7 ± 72.8	637.9 ± 78.9	201.7 ± 16.8	68.3 ± 14.2	31.4 ± 4.5	1.1 ± 0.2
	Percentage	83.8 ± 2.9	76.6 ± 2.3	56.6 ± 3.1	23.3 ± 2.3	30.1 ± 0.7	3.4 ± 0.2
Esters	Amount	3.6 ± 0.1	6.3 ± 1.9	12.0 ± 4.5	6.1 ± 1.0	2.1 ± 0.8	0.2 ± 0.0
	Percentage	0.7 ± 0.1	0.8 ± 0.2	3.4 ± 1.4	2.1 ± 0.1	2.0 ± 0.5	0.7 ± 0.1
Aldehydes	Amount	27.0 ± 0.5	75.0 ± 11.3	14.6 ± 5.0	38.4 ± 6.1	7.4 ± 2.2	0.3 ± 0.1
	Percentage	5.0 ± 0.7	8.9 ± 1.4	4.1 ± 1.5	13.1 ± 0.5	7.1 ± 1.4	0.9 ± 0.3
Alkanes	Amount	12.4 ± 3.0	51.3 ± 5.4	52.8 ± 4.2	107.1 ± 12.8	10.5 ± 1.1	9.1 ± 2.9
	Percentage	2.3 ± 0.4	6.1 ± 0.7	14.8 ± 1.1	36.8 ± 1.7	10.2 ± 1.6	27.4 ± 5.6
β-Diketone	Amount	1.7 ± 0.3	8.4 ± 0.8	25.8 ± 3.5	41.6 ± 7.3	37.2 ± 5.8	0.2 ± 0.2
	Percentage	0.3 ± 0.0	1 @ 0.1	7.3 ± 1.2	11.5 ± 1.4	35.6 ± 0.3	0.7 ± 0.4
OH-β-diketone	Amount	tr	3.8 ± 0.4	3.7 ± 0.1	2.6 ± 0.6	2.6 ± 0.8	tr
	Percentage	–	0.5 ± 0.0	1.1 ± 0.1	0.9 ± 0.1	2.5 ± 0.9	–
Not identified	Amount	37.7 ± 7.3	46.1 ± 8.7	38.0 ± 6.8	27.9 ± 3.8	11.4 ± 3.9	4.2 ± 1.0
	Percentage	7.1 ± 2.3	5.6 ± 1.6	10.6 ± 14.6	9.8 ± 2.7	10.7 ± 2.1	12.9 ± 2.2


*The wax loads of seedling leaves, flag leaves, leaf sheaths, and peduncles are shown as μg dm^-*2*^ surface area, and the wax loads of glumes and anther are expressed as μg g^-*1*^ fresh weight. Relative content (%) is also given for chemical classes in different organs. Each value is the mean ± SD from three biological replicates. tr, trace, values that were below 0.1 μg dm^-*2*^ or 0.1 μg g^-*1*^. Prim. alcohols, Primary alcohols.*

**FIGURE 2:**
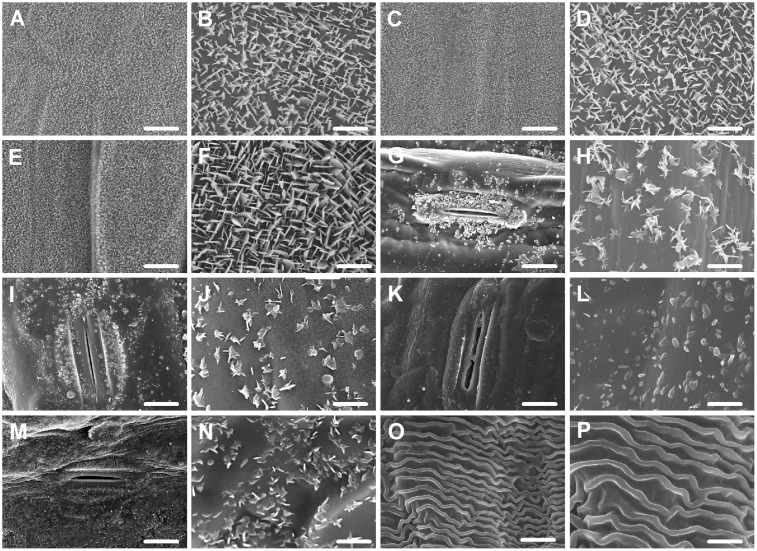
Epicuticular wax crystals on the surfaces of various organs in *Ae. tauschii* detected by scanning electron microscope (SEM). **(A,B)** Adaxial surface of seedling leaf. **(C,D)** Abaxial surface of seedling leaf. **(E,F)** Adaxial surface of flag leaf. **(G,H)** Abaxial surface of flag leaf. **(I,J)** Leaf sheaths. **(K,L)** Peduncles. **(M,N)** Glumes. **(O,P)** Anthers. **(A,C,E,G,I,K,M,O)** Were detected at 2,000× magnification, Scale bars = 10 μm. **(B,D,F,H,J,L,N,P)** Were detected at 10,000× magnification, Scale bars = 2 μm.

Since wax morphology is influenced by wax composition (Zhang et al., 2013), we investigated the morphology of wax crystals deposited in various organs by SEM. In seedling leaves, a dense array of wax platelets was found covering both adaxial and abaxial surfaces (**Figures 2A–D**), while in flag leaves, wax crystal density of both sides was different, with thicker platelet-shaped wax crystals being on adaxial surface (**Figures 2E,F**) and scattered platelet-shaped crystals being on abaxial surface (**Figures 2G,H**). Similarly, platelet-shaped crystals were found in leaf sheaths, peduncles, and glumes, but distributed sparsely (**Figures 2I–N**). In anthers, wave-shaped wax crystals were found (**Figures 2O,P**). These observations are consistent with studies showing that platelet-shaped wax crystals are associated with the deposition of primary alcohols in wheat leaf blades (Hallam and Chambers, 1970; Gülz, 1994).

Previous studies have shown that formation of primary alcohols was catalyzed by FAR in the acyl-reduction pathway (Kunst and Samuels, 2003; Samuels et al., 2008). Since high level of primary alcohols are present in *Ae. tauschii* leaves, FAR may play a significant role in the accumulation of leaf cuticular wax in *Ae. tauschii*. We thus continued to search for the genes encoding FARs in *Ae. tauschii*.

### Molecular Identification of *Ae.tFARs* from *Ae. tauschii* Leaf

Using the amino acid sequence of Arabidopsis CER4/FAR3 (GenBank accession no. NP567936) as a query, a BLAST search was performed in the *Ae. tauschii* genome database (NCBI BioProject ID: PRJNA182898). In total, 10 *FAR*-like genes (designated as *Ae.tFAR1* to *Ae.*t*FAR10*) were obtained, with amino acid sequences being 24–47% and 40–100% identical with Arabidopsis CER4 and wheat FARs over the entire length, respectively (Supplementary Tables 3, 5).

**FIGURE 3:**
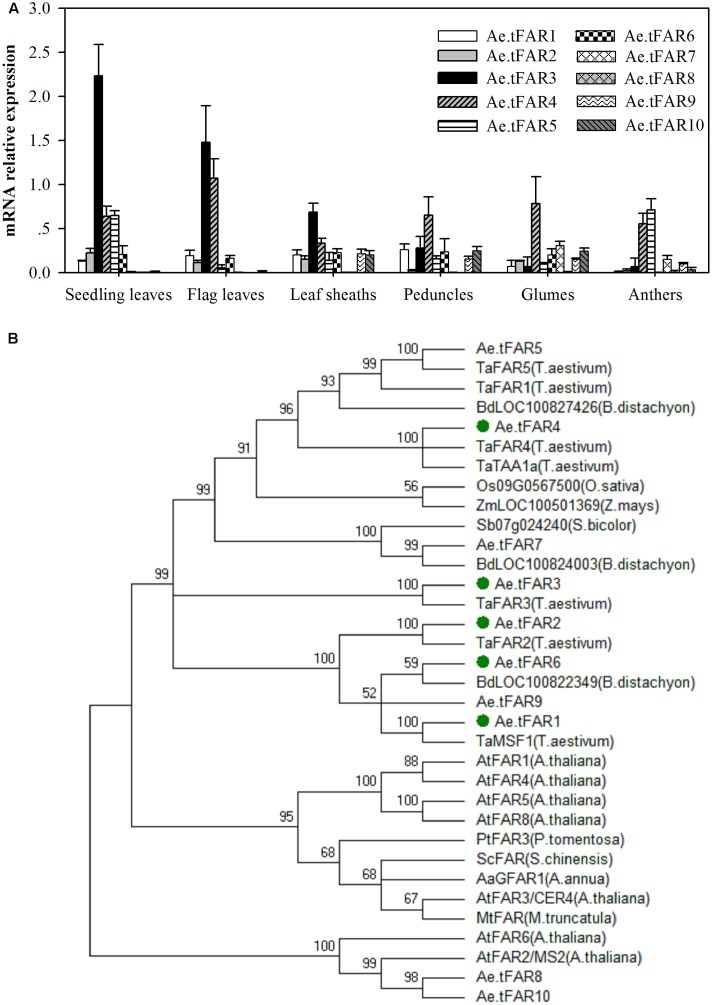
Gene expression and phylogenetic analysis of Ae.tFAR. **(A)** Tissue-specific gene expression patterns of *Ae.tFAR* by quantitative real-time PCR (qRT-PCR). Each value is the mean of three biological replicates. Error bars = SD. **(B)** Phylogenetic tree of the alcohol-forming fatty acyl-CoA reductases (FARs) from different species. The GenBank accession numbers of the FAR sequences from different plant species are summarized in Supplementary Table 4.

Tissue-specific expression patterns of these putative *Ae.tFAR* genes were determined by qRT-PCR using RNA derived from various organs, including seedling leaves, flag leaves, leaf sheaths, peduncles, glumes, and anthers (**Figure 3A**). The results showed that *Ae.tFAR3* was the most expressed gene in seedling leaves and flag leaves, while *Ae.tFAR5* was largely restricted to seedling leaves and anthers. *Ae.tFAR4* was found to be widely expressed in all aerial organs, whereas *Ae.tFAR7* to *Ae.tFAR10* genes were expressed only at later developmental stages of *Ae. tauschii. Ae.tFAR1*, *Ae.tFAR2*, and *Ae.tFAR6* transcripts were detected in all organs, but at low levels.

In order to identify FARs associated with biosynthesis of leaf cuticular wax, *FAR* genes expressed in leaves of *Ae. tauschii* were selected for further analysis. Of six genes (*Ae.tFAR1*–*6*) expressed in leaves, *Ae.tFAR5* was 100% identical with a known alcohol-forming FAR (TaFAR5) in hexaploid wheat (Wang et al., 2015c). Thus, we subsequently focused on the other five *FAR*-like genes (*Ae.tFAR1*–*4* and *Ae.tFAR6*) in *Ae. tauschii.* A phylogenetic analysis using neighbor-joining methods showed that the five Ae.tFARs were highly related to FARs from other species and belonged to the same monocot clade as wheat FARs (**Figure 3B**). These five Ae.tFARs were further analyzed to determine whether they are involved in the biosynthesis of primary alcohols in the leaf blades of *Ae. tauschii*.

### Analysis of *Ae.tFARs*

To isolate the five *FAR* candidates, reverse transcription-PCR (RT-PCR) was performed with sets of specific primers and templates that were derived from leaf blades of *Ae. tauschii*. Sequence analysis revealed that *Ae.tFAR1*, *Ae.tFAR2*, *Ae.tFAR3*, *Ae.tFAR4*, and *Ae.tFAR6* cDNA contained open reading frames of 1,497, 1,470, 1,497, 1,524, and 1500bp, encoding a polypeptide of 498, 489, 498, 507, and 499 amino acids, respectively. The molecular masses of Ae.tFAR1, Ae.tFAR2, Ae.tFAR3, Ae.tFAR4, and Ae.tFAR6 protein were predicted to be 57.0, 55.8, 55.7, 57.3, and 56.7 kDa, respectively.

**FIGURE 4:**
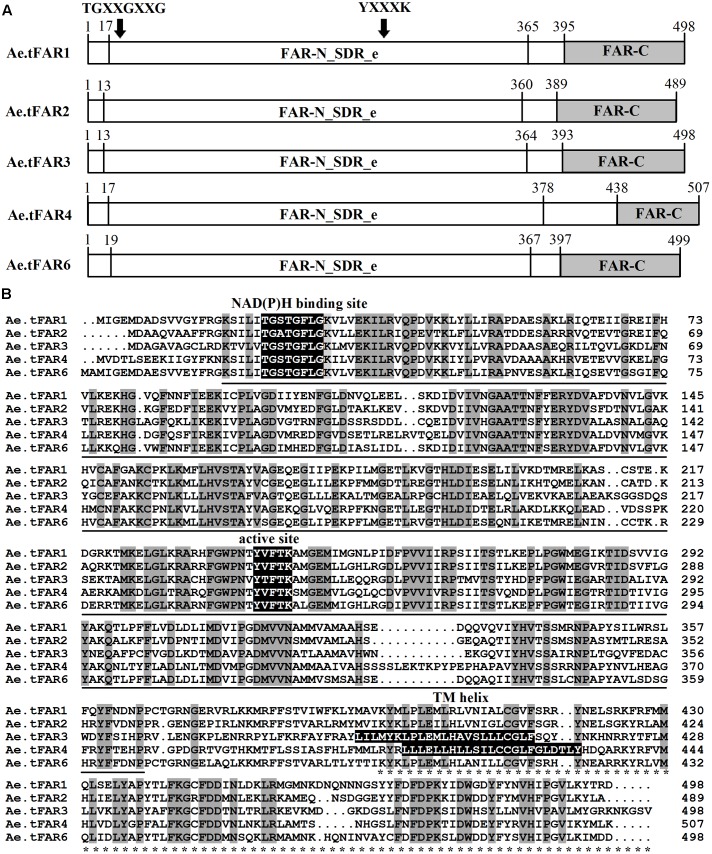
The structure and functional domains of the five Ae.tFAR proteins and amino acid sequence alignment. **(A)** Predicted functional domains of *Ae. tauschii* FARs. The conserved FAR-N_SDR_e and FAR_C domains are marked by underline and asterisks, respectively. The numbers indicate amino acid positions. **(B)** Amino acid sequences of the five Ae.tFARs. Identical amino acids are highlighted in dark gray. Three conserved motifs, NAD(P)H binding site motif (TGXXGXXG), active site motif (YXXXK), and TM (transmembrane) helix motif are highlighted in black (where X represents any amino acid).

A predictive protein analysis indicated that the five Ae.tFARs contain two distinct FAR protein domains: a Rossmann-fold NADB binding domain (FAR-N_SDR_e) linked with a FAR domain (FAR_C) (**Figure 4A**), in accordance with FAR proteins from other plant species, such as TaFAR1, TaFAR2, TaFAR3, and TaFAR4 in wheat (Wang et al., 2015b, 2016), and AtFAR5 and AtFAR8 in Arabidopsis (Chacón et al., 2013). Proteins with these two domains are believed to function as FARs, acting on medium- and long-chain fatty acids. Furthermore, the Rossmann-fold domain had two classical conserved motifs, a NAD(P)H binding site motif (TGXXGXXG) and an active site motif (YXXXK) (**Figure 4B**). Analysis by TMHMM program revealed that Ae.tFAR3 and Ae.tFAR4 contain one transmembrane helix between residues 391 to 413 and 411 to 433, respectively. This feature was found to be similar with that in jojoba FAR, which also contains membrane-spanning domains (Metz et al., 2000). However, no transmembrane domain was found in Ae.tFAR1, Ae.tFAR2, and Ae.tFAR6, which is similar to the alcohol-forming Arabidopsis FAR3/CER4 (Rowland et al., 2006, **Figure 4B**). Based on these analyses, we hypothesized that these five Ae.tFAR proteins might have the reductive activity of FAR and be involved in primary alcohol biosynthesis in leaf blades of *Ae. tauschii*.

### Heterologous Expression of Putative Ae.tFARs in Yeast

**FIGURE 5:**
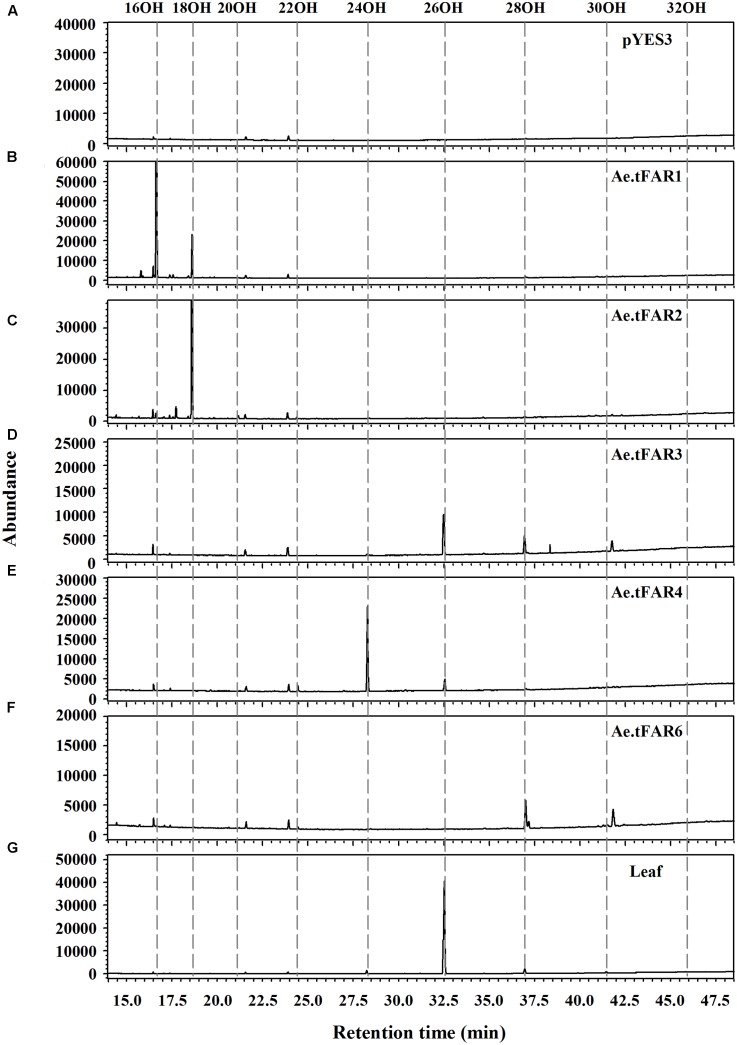
Heterologous expression of the five Ae.tFARs in yeast mutant INVSur4#. The yeast expressing **(A)** empty vector control pYES3 or vector harboring **(B)** *Ae.tFAR1* **(C)** *Ae.tFAR2*, **(D)** *Ae.tFAR3*, **(E)** *Ae.tFAR4* or **(F)** *Ae.tFAR6*. Transgenic yeast cells were grown in stringent medium lacking tryptophan and uracil. Primary alcohols were separated from other neutral lipids by thin layer chromatography (TLC). In empty vector control, no primary alcohols were detected. **(G)** All primary alcohols in transgenic yeast were also observed in the leaf blades of *Ae. tauschii*.

To prove that Ae.tFAR1, Ae.tFAR2, Ae.tFAR3, Ae.tFAR4, and Ae.tFAR6 are FARs responsible for the production of primary alcohols, we expressed the five *Ae.tFAR* genes in a yeast system. The open reading frames of the five *Ae.tFAR* genes were cloned into the yeast expression vector pYES3 downstream of the GAL1 promoter. Since C20 to C32 primary alcohols are components of leaf cuticular wax in *Ae. tauschii*, the presence of same chain lengths of VLCFA substrates in yeast is required. However, wild-type yeast can not produce VLCFAs of longer than C28, which may hamper us from correctly assessing the catalytic capacities of Ae.tFARs (Oh et al., 1997; Rowland et al., 2006; Domergue et al., 2010). To circumvent this problem, we used a yeast mutant INVSur#4 by introducing the vector p416 MET25-FLAG3:Sur4-F262A/K266L into wild-type yeast. This yeast mutant was capable of producing ≥C28 fatty acids (Denic and Weissman, 2007; Bernard et al., 2012, Supplementary Table 2). When Ae.tFARs were expressed in INVSur4#, all transgenic yeast cells produced novel chemical compounds (**Figure 5**), which were identified as primary alcohols ranging from C16 to C20 in yeast cells expressing Ae.tFAR1 and Ae.tFAR2 (**Figures 5B,C**), from C22 to C30 in yeast cells expressing Ae.tFAR3 and Ae.tFAR6 (**Figures 5D,F**), and from C22 to C26 in yeast cells expressing Ae.tFAR4 (**Figure 5E**). In contrast, yeast cells hosting empty vectors accumulated no primary alcohols (**Figure 5A**). It is noted that all these primary alcohols produced by Ae.tFARs in yeast were consistent with those detected in the leaf blades of *Ae. tauschii* in our study (**Figure 5G**).

Further analysis revealed that each Ae.tFAR showed distinct chain length preferences. Expression of Ae.tFAR1 resulted in large quantities of long-chain C16 alcohol (97.6%), and very small amounts of C18 (2.3%) and C20 (0.1%) alcohols. In contrast, expression of Ae.tFAR2 mainly produced C18:0-OH (99.2%), with traces of C16:0-OH (0.4%) and C20:0-OH (0.3%) being detected. It appeared that Ae.tFAR4 preferred producing C24:0-OH (86.8%) to C26:0-OH (10.4%) and C22:0-OH (2.8%). Ae.tFAR6 was primarily responsible for generating C28:0-OH (91.3%). For Ae.tFAR3, the major product was C26:0-OH (62.2%), followed by C28 (34.1%), C24 (1.8%), C30 (1.0%), and C22 (0.8%) alcohols (**Table 2**). These results confirmed that the five Ae.tFARs from *Ae. tauschii* are FAR, being able to produce long and very-long-chain primary alcohols. Notably, Ae.tFAR3 mainly produced C26 primary alcohol, which is the richest alcohol species in the leaf blades of *Ae. tauschii*.

**Table 2:** Chain length distributions of primary alcohols in yeast expressing Ae.tFARs.

Chain length	Empty vector	Ae.tFAR1	Ae.tFAR2	Ae.tFAR3	Ae.tFAR4	Ae.tFAR6
16:0-OH	n.d.	97.6 ± 0.2	0.4 ± 0.1	n.d.	n.d.	n.d.
18:0-OH	n.d.	2.3 ± 0.1	99.2 ± 0.1	n.d.	n.d.	n.d.
20:0-OH	n.d.	0.1 ± 0.1	0.3 ± 0.0	n.d.	n.d.	n.d.
22:0-OH	n.d.	n.d.	n.d.	0.8 ± 0.5	2.8 ± 0.7	4.0 ± 0.4
24:0-OH	n.d.	n.d.	n.d.	1.8 ± 0.1	86.8 ± 2.4	1.8 ± 0.4
26:0-OH	n.d.	n.d.	n.d.	62.2 ± 6.0	10.4 ± 1.8	1.3 ± 0.3
28:0-OH	n.d.	n.d.	n.d.	34.1 ± 5.6	n.d.	91.3 ± 1.0
30:0-OH	n.d.	n.d.	n.d.	1.0 ± 0.3	n.d.	1.6 ± 1.0
32:0-OH	n.d.	n.d.	n.d.	n.d.	n.d.	n.d.


*Relative content (%) is given for each chain length of primary alcohol in each Ae.tFAR-expressing yeast strain. Each value is the mean ± SD of three biological replicates. n.d., not detected.*

### Expression Levels and Chromosomal Localization of *Ae.tFAR* Paralogous Genes in Wheat

**FIGURE 6:**
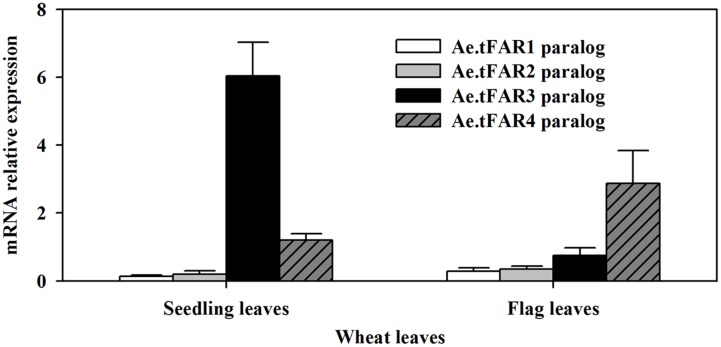
Expression analyses of *Ae.tFAR* paralogous genes in hexaploid wheat. The relative expression levels of *Ae.tFAR* paralogous genes were determined in seedling leaves and flag leaves of wheat (cv. Chinese Spring, CS) by qRT-PCR analysis. Each value is the mean of three independent parallel experiments. Error bars represent the SD.

In order to further confirm that the five Ae.tFARs are able to generate primary alcohols, we investigated the functions of *Ae.tFAR* paralogous genes in wheat. Ae.tFAR amino acid sequences were used as queries to search the wheat genome database (Ensembl Plants). Nine sequences in wheat D genome were found to be identical with Ae.tFAR amino sequences (Supplementary Table 3), while no sequence was found to match with Ae.tFAR6 amino acid sequence. The paralogous genes of Ae.tFAR1, Ae.tFAR2, Ae.tFAR3, Ae.tFAR4, Ae.tFAR5, Ae.tFAR7, Ae.tFAR8, Ae.tFAR9, and Ae.tFAR10 are located on wheat chromosome 3D, 7D, 5D, 4D, 4D, 7D, 1D, 3D, and 4D, respectively. We then examined the relative expression levels of these *Ae.tFAR* paralogous genes in seedling and flag leaves of wheat. The results showed that the *Ae.tFAR3* and *Ae.tFAR4* paralogs, Traes_5DL_5597A11EC.1 and Traes_4DL_9480F40CF.1, were highly expressed in wheat seedling leaf and flag leaves, respectively (**Figure 6**), highlighting the importance of *Ae.tFAR3* and *Ae.tFAR4* in the primary alcohol biosynthesis in the leaf blades.

**FIGURE 7:**
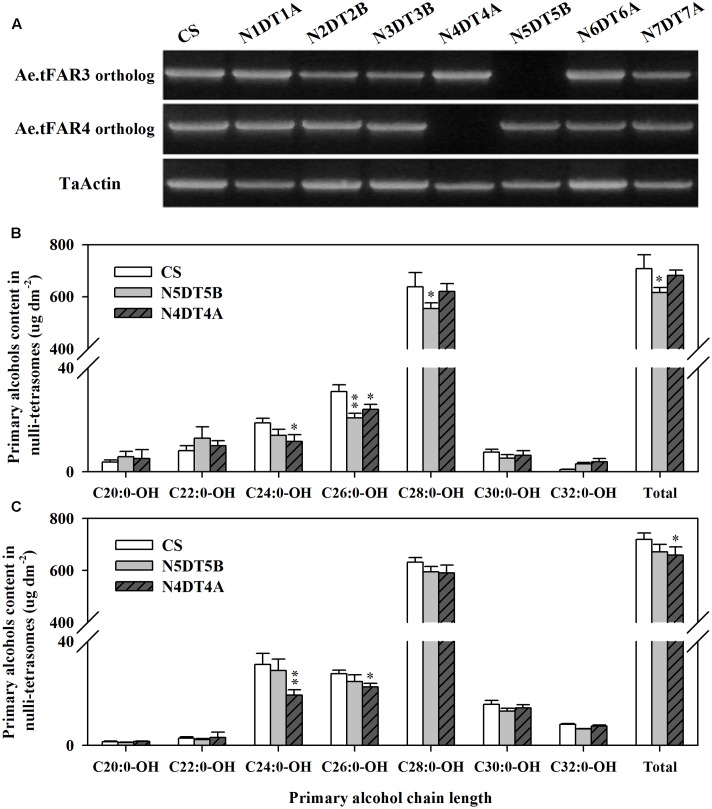
Chromosomal localization of *Ae.tFAR* paralogs and primary alcohol profiles of wheat nullisomic lines. **(A)** Chromosomal localization of *Ae.tFAR* paralogs in nullisomic–tetrasomic lines of wheat (cv. CS). N5DT5B and N4BT4A are abbreviations for nullisomic–tetrasomics of common wheat, namely nulli5D–tetra5B and nulli4B–tetra4A, respectively. The coverage and chain length distributions of primary alcohols from **(B)** seedling leaves and **(C)** flag leaves in CS and the nullisomic–tetrasomic lines. Each value is the mean of three independent measurements. Error bars indicate SD, and significant differences were assessed according to Student’s *t*-test (^∗^ for *P* < 0.05; ^∗∗^ for *P* < 0.01).

The wheat nullisomic–tetrasomic substitution lines were used to further investigate Ae.tFAR3 and Ae.tFAR4 paralogs in hexaploid wheat. cDNA was isolated from a set of seven nullisomic–tetrasomic substitution lines of wheat cv. CS with the loss of one D-chromosome and then was used as template for the amplification of *Ae.tFAR3* and *Ae.tFAR4* paralogs. As shown in **Figure 7A**, no amplification product was detected in N5DT5B and N4DT4A, which lacked the whole of chromosome 5D and 4D, respectively. In contrast, PCR products were obtained from all the other six nullisomic–tetrasomic substitution lines. Our results further confirm that *Ae.tFAR3* and *Ae.tFAR4* paralogs (Traes_5DL_5597A11EC.1 and Traes_4DL_9480F40CF.1) are located on wheat chromosome 5D and 4D. The substitution lines N5DT5B and N4DT4A lacking *Ae.tFAR3* and *Ae.tFAR4* paralogs, respectively, were then further analyzed. The wax mixtures from seedling and flag leaves of these substitution lines lacking of corresponding *Ae.tFAR3* and *Ae.tFAR4* paralogs showed a significant reduction in primary alcohols. Compared with wild-type wheat, the N5DT5B substitution line lacking of *Ae.tFAR3* paralog (Traes_5DL_5597A11EC.1) exhibited a large decrease in the amount of C26:0-OH and C28:0-OH primary alcohols in seedling leaf (**Figure 7B**), while N4DT4A with the loss of *Ae.tFAR4* paralog (Traes_4DL_9480F40CF.1) showed a notable decrease in C24:0-OH and C26:0-OH primary alcohols in seedling and flag leaves (**Figures 7B,C**). Altogether, these results might hint that *Ae.tFAR3* and *Ae.tFAR4* encode active FARs, catalyzing the biosynthesis of primary alcohols in the leaf blades of *Ae. tauschii*.

## Discussion

Fatty acyl-CoA reductase catalyzes the production of primary alcohols in the biosynthesis of cuticular wax in plants. In a previous work, we have identified 12 genes, which are located on D genome in wheat, are associated with wax biosynthesis (Wang et al., 2016). Of 12 genes, *TaFAR2* and *TaFAR5* are responsible for the biosynthesis of C18 and C22 primary alcohols, respectively (Wang et al., 2015c, 2016). However, knowledge on the rest of genes on D genome encoding alcohol-forming enzymes is still lacking. As *Ae. tauschii* contributes to the D-genome of bread wheat, study on wax biosynthesis in *Ae. tauschii* may provide insights on wax biosynthesis in bread wheat. In the present work, we identified and characterized five *Ae.tFARs* in leaves of *Ae. tauschii*, including *Ae.tFAR1*, *Ae.tFAR2*, *Ae.tFAR3*, *Ae.tFAR4*, and *Ae.tFAR6*. We have concluded that the five *Ae.tFARs* are involved in the biosynthesis of primary fatty alcohols in the leaf blades of *Ae. tauschii*. The evidence presented includes: (1) Gene expression patterns of *Ae.tFARs* are associated with deposition sites of primary alcohols. (2) Heterologous expression of Ae.tFAR1, Ae.tFAR2, Ae.tFAR3, Ae.tFAR4, and Ae.tFAR6 in yeast primarily produces C16:0-OH, C18:0-OH, C26:0-OH, C24:0-OH, and C28:0-OH, respectively. (3) The nullisomic–tetrasomic lines of hexaploid wheat lacking corresponding *Ae.tFAR* paralogous genes have significantly reduced levels of primary alcohols in the leaf blades.

Investigation of wax profiles from different organs of *Ae. tauschii* confirmed that primary alcohols are the dominant wax components in the leaf blades. Studies have shown that micromorphology and density of wax vary depending on wax composition and content of each component. It is proposed that platelet-like wax crystals are often associated with primary alcohols (Baker, 1982; Barthlott et al., 1998). In this study, the appearance of dense platelet-like crystals coincided with the high level of primary alcohols in the leaf blades of *Ae. tauschii*. When primary alcohols were much reduced in peduncle and glumes, the platelet-like crystals became scarce, further supporting the correlation between primary alcohol content and density of platelet-like crystals.

Like other FARs, the five Ae.tFARs possess Rossmann-fold NADB binding domain and FAR_C domain, and an active site YXXXK motif, indicating a catalytic role of these Ae.tFARs. Expression in yeast confirmed that the five Ae.tFARs produce primary alcohols ranging from C16:0 to C30:0. Ae.tFAR1, Ae.tFAR2, Ae.tFAR4, and Ae.tFAR6 are mainly responsible for generating C16:0-OH, C18:0-OH, C24:0-OH, and C28:0-OH, respectively. Interestingly, Ae.tFAR3 was most important for producing C26 primary alcohol and in the meanwhile, C26 primary alcohol was identified as the dominant wax component in the leaf blades of *Ae. tauschii*, suggesting the essential role of Ae.tFAR3 in wax accumulation of *Ae. tauschii*. To our best knowledge, the functions of these fatty alcohols-forming FARs have not yet been verified in *Ae. tauschii*. Arabidopsis CER4/FAR3 and wheat TaFAR1 and TaFAR5 are capable of producing C28 primary alcohol in plants, however, they are not able to generate primary alcohols beyond C26, at least, in yeast strains W3031A and INVSc1 (Rowland et al., 2006; Wang et al., 2015c, 2011). We speculate that the difference is due to the unavailability of substrates past C26 and/or the lack of substrate specificities of these FARs in yeast. In our experiment, we used INVSur4# to produce substrates with a wider range of chain lengths. Expression of Ae.tFAR3 and Ae.tFAR6 in INVSur4# resulted in the production of primary alcohols, with chain lengths being C26 or longer than C26. In contrast, three other Ae.tFARs (Ae.tFAR1, Ae.tFAR2, and Ae.tFAR4) were still unable to make primary alcohols longer than C26 even when substrates of chain length long enough were available, indicating that Ae.tFAR1, Ae.tFAR2, and Ae.tFAR4 are incapable of catalyzing the synthesis of >C26 primary alcohols in yeast. It appears that FARs can catalyze the production of a few chain lengths of primary alcohols in *Ae. tauschii*, however, each one shows strong preference for one major chain length of substrate and thus produces one major product. For example, Ae.tFAR3 primarily produces C26 primary alcohol, while Ae.tFAR4 principally generates C24 primary alcohol, reflecting the distinct substrate specificities of Ae.tFARs with respect to acyl chain length. However, it is unknown so far what determines chain length specificities of Ae.tFARs in *Ae. tauschii*. A prior analysis of FAR8 in Arabidopsis has shown that chain length specificity is associated with several amino acids at the junction of the Rossmann-fold NADB binding domain and the FAR_C domain (Chacón et al., 2013). Whether this is also the case for Ae.tFARs merits further investigation.

It is recognized that *Ae. tauschii* is the D-genome donor to hexaploid wheat. Nine *Ae.tFAR* paralogous genes were found locating on D genome of hexaploid wheat. The nullisomic–tetrasomic substitution lines of wheat were used in this study to further validate the role of *Ae.tFARs* in the biosynthesis of primary alcohols. One wheat gene (Traes_5DL_5597A11EC.1) identical to *Ae.tFAR3* is located on 5D genome and the loss of 5D in the nullisomic–tetrasomic substitution line N5DT5B led to an obvious reduction in the amounts of primary alcohols in wheat leaves. Another wheat gene (Traes_4DL_9480F40CF.1) sharing 99% identity with *Ae.tFAR4* is located on 4D genome in wheat. Examination on the nullisomic–tetrasomic substitution line N4DT4A lacking of 4D genome revealed a substantial decrease in the contents of primary alcohols in flag leaves. The analysis of these wheat genes, which share 40–100% identity to *Ae.tFARs*, further supports that *Ae.tFAR3* and *Ae.tFAR4* are involved in the biosynthesis of primary alcohols.

The expression patterns of *Ae.tFARs* are consistent with their roles in the biosynthesis of primary alcohols in *Ae. tauschii*. The transcripts of *Ae.tFAR3*, *Ae.tFAR4*, and *Ae.tFAR6* were all found in leaves, in keeping with their functions in the production of C20 to C30 primary alcohols, which are components of leaf cuticular wax in *Ae. tauschii*. The highest level of expression was observed for *Ae.tFAR3* in the leaf blades, agreeing with the highest accumulation of C26 primary alcohol. In addition to wax biosynthesis, *FAR* genes have been reported contributing to the biosynthesis of suberin, cutin, and sporopollenin in plants (Metz et al., 2000; Wang et al., 2002; Pollard et al., 2008; Domergue et al., 2010; Beisson et al., 2012). The expression levels of *Ae.tFAR1* and *Ae.tFAR2* were low in leaves, and *Ae.tFAR1* and *Ae.tFAR2* were mainly involved in the generation of C16 and C18 primary alcohols, respectively. It is thus possible that these two genes function in the biosynthesis of suberin- and sporopollenin-associated primary alcohols in *Ae. Tauschii*. However, further evidence is required to draw a conclusion regarding the role of *Ae.tFAR1* and *Ae.tFAR2* in *Ae. tauschii*.

In summary, we have cloned *Ae.tFAR1*, *Ae.tFAR2*, *Ae.tFAR3*, *Ae.tFAR4*, and *Ae.tFAR6* from *Ae. tauschii* and provided evidence that *Ae.tFAR3*, *Ae.tFAR4*, and *Ae.tFAR6* encode FARs, which are involved in the production of wax-related primary alcohols. Particularly, *Ae.tFAR3* is mainly responsible for generating C26:0-OH in the leaf blades of *Ae. tauschii* where C26 primary alcohol is also the dominant wax component.

## Author Contributions

MW, YW, and ZW contributed to the experimental design. MW, HW, JX, CL, and YW performed the experiments and analyzed the data. MW wrote the manuscript and ZW revised it.

## Conflict of Interest Statement

The authors declare that the research was conducted in the absence of any commercial or financial relationships that could be construed as a potential conflict of interest.
