# Supplementary material for: Five Fatty Acyl-Coenzyme A Reductases Are Involved in the Biosynthesis of Primary Alcohols in Aegilops tauschii Leaves
Source: Front Plant Sci. 2017 Jun 12;8:1012. doi: 10.3389/fpls.2017.01012 (PMC5466989; doi:10.3389/fpls.2017.01012)
Supplement: Supplementary file 1 [file Table_1.DOCX]

**Supplementary Table 1** Sequences of primers used for PCR reactions. Restriction enzyme sites are bold.

| **Genes** | **Forward primer (5' 3')** | **Reverse primer (5' 3')** |
| --- | --- | --- |
| Primers used for gateway cloning of cDNAs or histidine mutated cDNAs | | |
| Ae.tFAR1 | ATGATCGGTGAAATGGATGCG | TCAATCACGCGTGTATTTGAGC |
| Ae.tFAR2 | ATGGATGCTGCTCAGGTCGC | TCACGCCAGATACTTGAGCAC |
| Ae.tFAR3 | ATGGACGCTGGCGCGGTGGC | CTATACGCTTCCCTTATTTTTGCG |
| Ae.tFAR4 | ATGGTTGACACACTGAGTGAAGAG | TCACTTGAGCATGTACTTTATGACC |
| Ae.tFAR6 | ATGGCGATGATCGGTGAAAT | TTAATCATCCATAATTTTTAGCAC |
| Primers used for quantitative real-time PCR analysis in *Ae. tauschii* and wheat | | |
| Ae.tFAR1 | ACTGAGATCATAGGTCGGGAG | TTCCTCGAGCTGGACATTG |
| Ae.tFAR1/ortholog | ATGGGCGAGACCCTAAAGGT | CATGGTTTTCCTCCCATCCT |
| Ae.tFAR2/ortholog | TCTGGTAAACATTGGGCTATG | CCTCCATCACTATTTTGCTCC |
| Ae.tFAR3/ortholog | CATGTGAGCTCGGCAATCC | ATTTTCTAGTACACGGGGATGG |
| Ae.tFAR4/ortholog | CATTGGAGAAGACAAAGCCATAT | ATGTCATCTTATGGGTACCCACG |
| Ae.tFAR5/ortholog | CCTCCACTCGGACATCAGAG | TCGGTACAACACCGCGTAC |
| Ae.tFAR6 | TCAGAGGTTACCGGGAGCG | ATCTATCAGGCTGGCAATGT |
| Ae.tFAR7 | ATGTCGAGCTCGTCCAATTC | CCACGGAGTTTGCTGGAAT |
| Ae.tFAR8 | TAGCTTGGGAGACACCATCA | CATTTCTTGAGAGAAAGATGCTG |
| Ae.tFAR9 | ATGATCGCTGGAATGGATGCGG | CTGGCTGAACCCTCAGTATCTTC |
| Ae.tFAR10 | GTCAACCTCAACAAGCTTAGGC | GCATCACTTGAGGACGTACTTC |
| Ae.tGAPDH | GAGGTGCACTGCAGTCAAAGT | GCATCCATTACCAAGCCTTC |
| TaActin | GAGCTATGAGATGCCTGATGGT | CGCTTCGTGTTACCAGGAACT |
| Primers used for yeast expression | | |
| Ae.tFAR1 | CGG**GGTACC**AAAAAAATGTCTATGATCGGTGAAATGGATG | ATAAGAAT**GCGGCCGC**TCAATCACGCGTGTATTTGAGC |
| Ae.tFAR2 | AACC**GGAATTC**ATGGATGCTGCTCAGGTCGC | CCG**CTCGAG**TCACGCCAGATACTTGAGCAC |
| Ae.tFAR3 | CGG**GGTACC**AAAAAAATGTCTATGGACGCTGGCGCGGTGG | ATAAGAAT**GCGGCCGCC**TATACGCTTCCCTTATTTTTGC |
| Ae.tFAR4 | CCC**AAGCTT**AAAAAAATGTCTATGGTTGACACACTGAGT | AACCG**GAATTC**TCACTTGAGCATGTACTTTATGACC |
| Ae.tFAR6 | CGG**GGTACC**AAAAAAATGTCTATGGCGATGATCGGTGAAAT | ATAAGAAT**GCGGCCGC**TTAATCATCCATAATTTTTAGC |
| Primers used for chromosomal localization of orthologs analysis in wheat | | |
| Ae.tFAR3 ortholog | AGGTTTGTCGATTCTCAATCCTT | GGAACTATCTTCTCTTTGATCAGC |
| Ae.tFAR4 ortholog | ACACCTTCATGGCCCGTGTAT | CCTCAGTCTCCACCCGATGCTT |
