# Supplementary material for: Five Fatty Acyl-Coenzyme A Reductases Are Involved in the Biosynthesis of Primary Alcohols in Aegilops tauschii Leaves
Source: Front Plant Sci. 2017 Jun 12;8:1012. doi: 10.3389/fpls.2017.01012 (PMC5466989; doi:10.3389/fpls.2017.01012)
Supplement: Supplementary file 2 [file Table_2.DOCX]

**Supplementary Table 2** Transgenic yeasts. The vector p416 MET25-FLAG3:Sur4-F262A/K266L was co-transformed with empty vector pYES3 or with vector harboring Ae.tFARx into the yeast INVSc1 cells.

| **Genes expressed** | **Expression vectors** | | **Selection medium** |
| --- | --- | --- | --- |
| SUR4# + empty vector | pYES3 | p416 MET25-FLAG3:Sur4-F262A/K266L | -TRP-URA |
| SUR4# + Ae.tFAR1 | pYES3:Ae.tFAR1 | p416 MET25-FLAG3:Sur4-F262A/K266L | -TRP-URA |
| SUR4# + Ae.tFAR2 | pYES3:Ae.tFAR2 | p416 MET25-FLAG3:Sur4-F262A/K266L | -TRP-URA |
| SUR4# + Ae.tFAR3 | pYES3:Ae.tFAR3 | p416 MET25-FLAG3:Sur4-F262A/K266L | -TRP-URA |
| SUR4# + Ae.tFAR4 | pYES3:Ae.tFAR4 | p416 MET25-FLAG3:Sur4-F262A/K266L | -TRP-URA |
| SUR4# + Ae.tFAR6 | pYES3:Ae.tFAR6 | p416 MET25-FLAG3:Sur4-F262A/K266L | -TRP-URA |
