# Supplementary material for: Five Fatty Acyl-Coenzyme A Reductases Are Involved in the Biosynthesis of Primary Alcohols in Aegilops tauschii Leaves
Source: Front Plant Sci. 2017 Jun 12;8:1012. doi: 10.3389/fpls.2017.01012 (PMC5466989; doi:10.3389/fpls.2017.01012)
Supplement: Supplementary file 3 [file Table_3.DOCX]

**Supplementary Table 3** Colinearity between *Aegilops tauschii* and *Triticum aestivum* in predicted FAR genes.

| **Gene** | **Aegilops tauschii** | **Sequence** | **Protein** | **Triticum aestivum** | **Identity** |
| --- | --- | --- | --- | --- | --- |
| **names** | **gene** | **length (bp)** | **ID** | **gene** | **%** |
| Ae.tFAR1 | KT692976 | 1497 | AMH86041 | Traes_3DS_71BF6EC00.1 | 43.17 |
| Ae.tFAR2 | F775_26985 | 1470 | M8B4B3 | Traes_7DS_A3D9FFE80.1 | 80.57 |
| Ae.tFAR3 | F775_14906 | 1497 | M8BJ01 | Traes_5DL_5597A11EC.1 | 99.40 |
| Ae.tFAR4 | F775_16212 | 1524 | M8CRK2 | Traes_4DL_9480F40CF.1 | 99.01 |
| Ae.tFAR5 | F775_30161 | 1569 | M8CCP2 | Traes_4DS_849C911C9.1 | 100 |
| Ae.tFAR6 | F775_20045 | 1500 | M8C929 | No match |  |
| Ae.tFAR7 | F775_15771 | 1545 | R7WFR5 | Traes_7DS_93F9ED9BE.2 | 39.78 |
| Ae.tFAR8 | F775_11380 | 1533 | M8BY01 | Traes_1DS_C91B806B6.1 | 63.21 |
| Ae.tFAR9 | F775_00666 | 1539 | R7VYK3 | Traes_3DS_44B20436F.1 | 87.70 |
| Ae.tFAR10 | F775_11143 | 1779 | M8CTW5 | Traes_4DL_021634BC0.1 | 81.21 |
