# Supplementary material for: Five Fatty Acyl-Coenzyme A Reductases Are Involved in the Biosynthesis of Primary Alcohols in Aegilops tauschii Leaves
Source: Front Plant Sci. 2017 Jun 12;8:1012. doi: 10.3389/fpls.2017.01012 (PMC5466989; doi:10.3389/fpls.2017.01012)
Supplement: Supplementary file 4 [file Table_4.DOCX]

**Supplementary Table 4** The accession numbers of *FAR* genes in phylogenetic analysis.

| **Gene names** | **Species** | **Accession no.** |
| --- | --- | --- |
| TaFAR1 | Triticum aestivum | KF926683 |
| TaFAR2 | Triticum aestivum | KJ675403 |
| TaFAR3 | Triticum aestivum | KT963076 |
| TaFAR4 | Triticum aestivum | KT963077 |
| TaFAR5 | Triticum aestivum | KJ725345 |
| TaTAA1a | Triticum aestivum | CAD30692 |
| Ta MSF1 | Triticum aestivum | CBI75514 |
| BdLOC100827426 | Brachypodium distachyon | XP_003578677 |
| BdLOC100824003 | Brachypodium distachyon | XP_003574925 |
| BdLOC100822349 | Brachypodium distachyon | XP_003573673 |
| Sb07g024240 | Sorghum bicolor | XP_002445686 |
| Os09g0567500 | Oryza sativa | NP_001063962 |
| ZmLOC100501369 | Zea mays | NP_001183038 |
| ScFAR | Simmondsia chinensis | AAD38039 |
| AtFAR1 | Arabidopsis thaliana | NP_197642 |
| AtFAR2/MS2 | Arabidopsis thaliana | ABZ10952 |
| AtFAR3/CER4 | Arabidopsis thaliana | NP_567936 |
| AtFAR4 | Arabidopsis thaliana | NP_190040 |
| AtFAR5 | Arabidopsis thaliana | NP_190041 |
| AtFAR6 | Arabidopsis thaliana | NP_191229 |
| AtFAR8 | Arabidopsis thaliana | NP_190042 |
| PtFAR3 | Populus tomentosa | AEV53412 |
| MtFAR | Medicago truncatula | XP_003596783 |
| AaGFAR1 | Artemisia annua | ADK66305 |
