# Supplementary material for: Five Fatty Acyl-Coenzyme A Reductases Are Involved in the Biosynthesis of Primary Alcohols in Aegilops tauschii Leaves
Source: Front Plant Sci. 2017 Jun 12;8:1012. doi: 10.3389/fpls.2017.01012 (PMC5466989; doi:10.3389/fpls.2017.01012)
Supplement: Supplementary file 5 [file Table_5.DOCX]

**Supplementary Table 5** Identity of ten AetFARs.

|  | Ae.tFAR1 | Ae.tFAR2 | Ae.tFAR3 | Ae.tFAR4 | Ae.tFAR5 | Ae.tFAR6 | Ae.tFAR7 | Ae.tFAR8 | Ae.tFAR9 | Ae.tFAR10 |
| --- | --- | --- | --- | --- | --- | --- | --- | --- | --- | --- |
| Ae.tFAR1 | - | 69.88 | 50.89 | 57.5 | 52.85 | 76.8 | 41.81 | 24.75 | 73.88 | 33.89 |
| Ae.tFAR2 | - | - | 50.5 | 55.29 | 52.2 | 70.14 | 41.85 | 25.33 | 67.19 | 33.78 |
| Ae.tFAR3 | - | - | - | 51.06 | 47.17 | 49.8 | 3.38 | 24.71 | 50.78 | 30.19 |
| Ae.tFAR4 | - | - | - |  | 75.1 | 56.42 | 46.63 | 25.53 | 53.51 | 32.34 |
| Ae.tFAR5 | - | - | - | - | - | 52.18 | 43.09 | 24.16 | 50.19 | 30.27 |
| Ae.tFAR6 | - | - | - | - | - | - | 43.05 | 23.72 | 72.96 | 31.93 |
| Ae.tFAR7 | - | - | - | - | - | - | - | 9.42 | 40.69 | 25.42 |
| Ae.tFAR8 | - | - | - | - | - | - | - | - | 25.12 | 43.67 |
| Ae.tFAR9 | - | - | - | - | - | - | - | - | - | 32.38 |
